# MicroRNA-199a acts as a potential suppressor of cardiomyocyte autophagy through targeting *Hspa5*

**DOI:** 10.18632/oncotarget.19133

**Published:** 2017-07-10

**Authors:** Liang Chen, Fei-Yu Wang, Zhen-Yu Zeng, Ling Cui, Jian Shen, Xiao-Wei Song, Pan Li, Xian-Xian Zhao, Yong-Wen Qin

**Affiliations:** ^1^ Department of Cardiology, Changhai Hospital, Second Military Medical University, Shanghai 200433, China; ^2^ Department of Laboratory Medicine, Changhai Hospital, Second Military Medical University, Shanghai 200433, China; ^3^ Department of Cardiology, People's Hospital of Inner Mongolia, Hohhot 010017, China; ^4^ Department of Cardiology, 411 Hospital of PLA Navy, Shanghai 200081, China

**Keywords:** microRNA, cardiomyocyte, autophagy, Hspa5

## Abstract

Autophagy is an adaptive response to cardiomyocytes survival under stress conditions. MicroRNAs (miRNAs, miR) have been described to act as potent modulators of autophagy. To investigate whether and how miR-199a modulated autophagy *in vitro*, primary cardiomyocytes were treated under starvation to induce autophagy. Results showed that down-regulation of miR-199a was sufficient to activate cardiomyocytes autophagy. MiR-199a suppressed cardiomyocytes autophagy through direct inhibiting heat shock protein family A member 5 (*Hspa5*). Forced overexpression of *Hspa5* recovered the inhibitory effect of miR-199a in autophagy activation. Our results suggested miR-199a as an effective suppressor of starvation-induced cardiomyocytes autophagy and that *Hspa5* was a direct target during this process. These results extend the understanding of the role and pathway of miR-199a in cardiomyocytes autophagy, and may introduce a potential therapeutic strategy for the protection of cardiomyocytes in myocardial infarction or ischemic heart disease.

## INTRODUCTION

Macroautophagy (hereafter referred to as autophagy) is a tightly regulated intracellular catabolic pathway involving the lysosomal degradation of long-lasting proteins and nonfunctional organelles, thereby generating fatty acids and amino acids that are used for mitochondrial adenosine triphosphate production [1]. During acute myocardial infarction, cardiomyocytes (CMs) suffer a state of nutrient deprivation [2]. Autophagy may serve to maintain energy production in response to acute ischemia and promote CMs survival [2, 3].

The process of autophagy is regulated by several signaling pathways, and important ties exist between microRNAs (miRNAs, miR) and the core autophagy machinery [4]. As endogenous non-coding RNA molecules, miRNAs play critical roles in a broad range of biological processes, linking them to numerous human diseases including cardiovascular diseases [4–6].

Recently, the physiological importance of miRNA-autophagy interconnection has been elucidated. Zhu et al. [7] first reported the link between miRNAs and autophagy, showing that miR-30a suppressed autophagy by inhibiting *BECN1*. Xiao et al. [8] first identified miR-204 as an autophagy regulator in CMs. Recent studies reported that several miRNAs could regulate CMs autophagy during different cardiac disorders [9–13]. However, considering the widespread importance of both miRNAs and autophagy in stress response and disease, it is intriguing but far from complete to understand these interactions.

MicroRNA-199a, which predominantly expresses in CMs, has been shown to relate to several cardiovascular disorders. We previously identified miR-199a as a regulator of cardiac hypertrophy [14]. In this study, we investigated the expression and role of miR-199a in CMs autophagy under starvation stress. We also demonstrated that miR-199a could suppress starvation-induced autophagy by inhibiting heat shock protein family A member 5 (*Hspa5*).

## RESULTS

### Starvation induces autophagy activation in CMs

**Figure 1:**
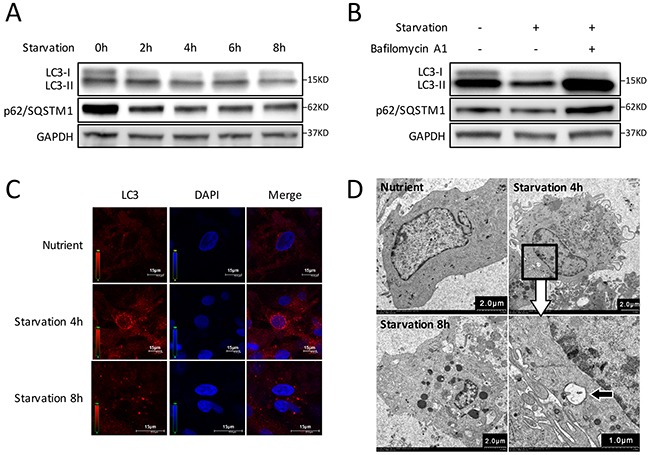
Autophagy is induced by starvation with EBSS in CMs **(A)** Time course of autophagy activation stimulated by starvation. Western blot shows the LC3-I decreased while LC3-II increased during the first 4 hours and gradually recovered after starvation for 6 h. The degradation of p62/SQSTM1 increased gradually in the first 4 hours, then decreased gradually. **(B)** CMs were treated with Bafilomycin A1 and starved for 4 h. LC3-II accumulation was markedly increased, and p62/SQSTM1 degradation was blocked in Bafilomycin A1-treated groups. GAPDH was used as endogenous control. **(C)** The intracellular autophagosome and autolysosomes at 4 h starvation were probed by LC3 dots (red) for cell immunofluorescence. Nuclei were stained with DAPI (blue). **(D)** Transmission electron microscopy showed double-membraned autophagosomes formed in CMs at 4 h starvation (black arrow).

Primary CMs were isolated from neonatal Sprague-Dawley rats (Supplementary Figure 1) and a starvation-induced CMs autophagy model with Earle's Balanced Salt solution (EBSS) was utilized [15, 16]. It is well known that conversion of microtubule-associated protein 1 light chain 3 (LC3) -I to LC3-II is a key step in autophagosome formation and a commonly used experimental marker of autophagy in mammals [15, 17]. In addition to LC3, decreased p62/SQSTM1 levels that are associated with autophagy activation was also employed [15]. We then detected the LC3 and p62/SQSTM1 levels by western blot to determine the autophagy activation and optimal stimulation time points of CMs. The results showed that, with prolongation of starvation, there was a trend that both LC3-II accumulation and p62/SQSTM1 degradation increased during the first 4 hours (h), and then decreased gradually (Figure 1A). Since both autophagosome formation and lessening in autophagosome clearance can lead to the increase of LC3-II, to discriminate between these two possibilities, we used Bafilomycin A1 which is a fusion inhibitor of autophagosomes and lysosomes, to inhibit the degradation of LC3-II and p62/SQSTM1. After 4 h starvation, LC3-II and p62/SQSTM1 levels were remarkably increased in Bafilomycin A1-treated CMs (Figure 1B). Furthermore, the number of fluorescent LC3 dots increased in 4 h starvation group, indicating accumulation of autophagosomes [15] and induction of autophagy in CMs (Figure 1C). We also assessed the double-membrane autophagosomes using TEM (Figure 1D). The above results indicate that autophagy was significantly induced in CMs after 4 h starvation stress.

### MiR-199a is down-regulated during starvation-induced CMs autophagy

**Figure 2:**
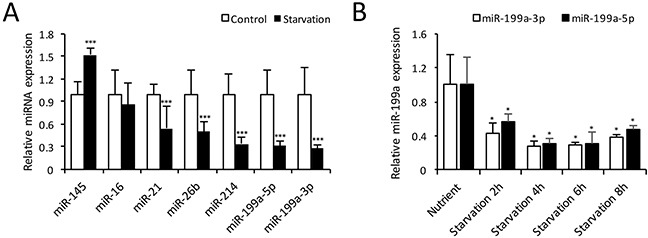
Expression of microRNAs in autophagic CMs **(A)** Relative miRNA expression levels at 4 h starvation compared with control group. The expression levels of miR-199a-3p, miR-199a-5p, miR-21, miR-26b and miR-214 were decreased significantly. The relative miR-145 expression was increased, while miR-16 showed no significant change. The differences between two groups were analyzed using *t*-tests. (***P<0.001, n=3). **(B)** Cardiomyocytes were treated with EBSS for 2-8 h, qRT-PCR showed both miR-199a-3p and 199a-5p expressions were decreased significantly from 2 h up to 8 h. *Dunnett's t* tests were used for comparison between groups. (*P<0.05 versus nutrient group, n=3). *U6* small nuclear RNA was used as reference.

As previously reported, a collection of miRNAs, including miR-16 [18], miR-21 [19], miR-26b [20], miR-145 [10], miR-199a [14] and miR-214 [21], were aberrantly expressed in cardiovascular diseases. We used qRT-PCR to investigate the expressions of these miRNAs during CMs autophagy induced by starvation. Compared with the nutrient group, the expressions of miR-199a-3p and miR-199a-5p decreased most significantly in CMs after 4 h starvation (Figure 2A). Subsequently, we examined the time course of miR-199a expression during starvation-induced autophagy. As shown in Figure 2B, the expression levels of miR-199a-3p and miR-199a-5p were both down-regulated during 2 to 8 h starvation and most pronounced at 4 h. These results indicate that miR-199a is down-regulated obviously during starvation-induced CMs autophagy.

### MiR-199a overexpression suppresses starvation-induced autophagy in CMs

**Figure 3:**
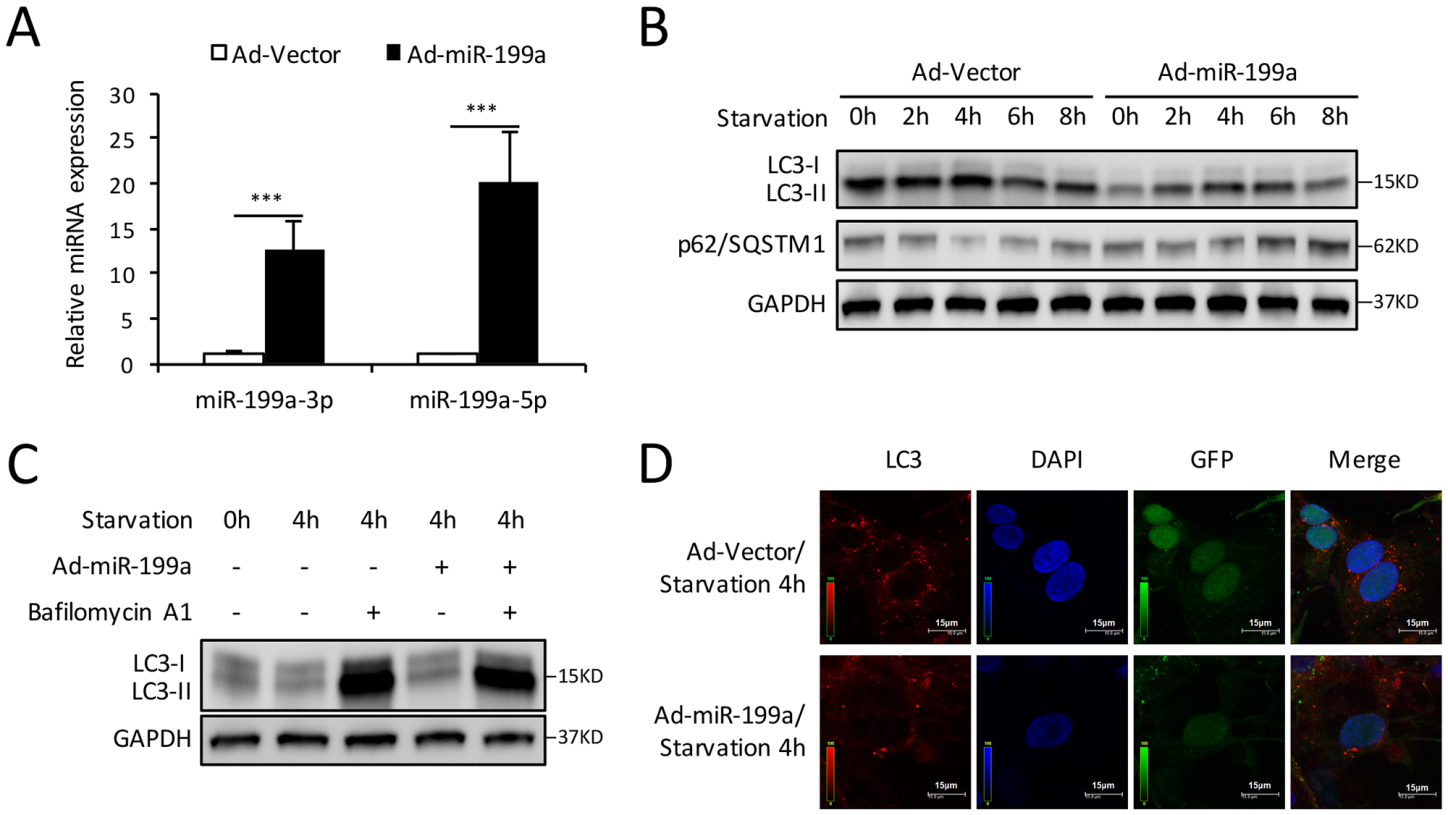
Overexpression of miR-199a blocks the autophagy activity in CMs **(A)** After transfection with miR-199a overexpression adenovirus (Ad-miR-199a), the miR-199a-3p and miR-199a-5p expression levels were markedly up-regulated compared with cells infected with Ad-vector. The differences between two groups were analyzed using *t*-tests. (***P<0.001 versus Ad-vector, n=3) **(B)** Western blot showed that overexpression of miR-199a significantly suppressed LC3-II accumulation and p62/SQSTM1 degradation at starvation for 2-8 h. **(C)** After 48 h transfection, CMs were treated with Bafilomycin A1 and exposed to starvation for 4 h. Western blot shows reduced LC3-II levels in miR-199a overexpression CMs treated with or without Bafilomycin A1. GAPDH was used as endogenous control. **(D)** MiR-199a blocked starvation-induced autophagosomes formation. At 4 h starvation, confocal microscopy showed that the number of autophagosomes as represented by LC3 dots (red) was reduced in Ad-miR-199a group compared with Ad-vector group. Nuclei were stained with DAPI (blue). GFP: green fluorescent protein fused in adenovirus.

To further study the function of miR-199a in CMs autophagy, an adenovirus expressing miR-199a (Ad-miR-199a) was generated with the adenovirus vector (Ad-vector) as control. QRT-PCR results showed that infection with Ad-miR-199a prominently increased miR-199a-3p and miR-199a-5p expression in CMs (Figure 3A). Then CMs were starved for 0-8 h and proteins were collected for western blot. Results showed that LC3-II accumulation and p62/SQSTM1 degradation reduced significantly after miR-199a overexpression (Figure 3B). In the presence of Bafilomycin A1, the LC3-II accumulation was still suppressed by miR-199a overexpression (Figure 3C), indicating that miR-199a may contribute to the formation of autophagosome. The immunofluorescence analysis also demonstrated that under the same starvation condition, the amount of red LC3 dots in Ad-miR-199a group was fewer than control (Figure 3D). These results indicate that overexpression of miR-199a suppresses starvation-induced autophagy in CMs.

### Knockdown of miR-199a promotes starvation-induced CMs autophagy

**Figure 4:**
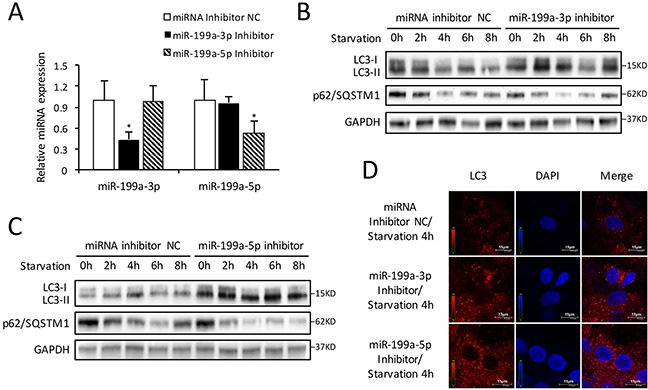
Inhibition of endogenous miR-199a stimulates autophagic activity **(A)** After transfection with miR-199a-3p inhibitor, the miR-199a-3p expression was suppressed while miR-199a-5p showed no significant change. Conversely, after transfection with miR-199a-5p inhibitor, the miR-199a-5p expression was decreased while miR-199a-3p showed no significant change. The analysis of variance for multi-group comparison was using *ANOVA* (*P<0.05 versus miRNA inhibitor NC, n=3). **(B** and **C)** Knockdown of either miR-199a-3p or miR-199a-5p stimulated LC3-II accumulation and p62/SQSTM1 degradation in CMs. GAPDH was used as endogenous control. **(D)** The results of immunofluorescence suggest that at 4 h starvation, knockdown of either miR-199a-3p or miR-199a-5p could increase the number of LC3 fluorescent dots (red) which indicated autophagosomes formation. Nuclei were stained with DAPI (blue).

To illuminate comprehensive function of miR-199a on autophagy, CMs were pretreated with miR-199a-3p or miR-199a-5p inhibitor in starvation-induced autophagy. QRT-PCR confirmed the down-regulation of miR-199a-3p or miR-199a-5p in CMs transfected with miR-199a inhibitor (Figure 4A). Western blot demonstrated that LC3-II accumulation and p62/SQSTM1 degradation enhanced after knockdown of either miR-199a-3p or miR-199a-5p (Figure 4B, 4C). In addition, fluorescence microscopic examination showed more red LC3 dots in CMs treated by miR-199a inhibitor comparing with miRNA inhibitor negative control group (Figure 4D). These findings suggest that down-regulation of either miR-199a-3p or miR-199a-5p could activate starvation-induced CMs autophagy.

### MiR-199a directly inhibits endoplasmic reticulum molecular chaperone gene *Hspa5*

**Figure 5:**
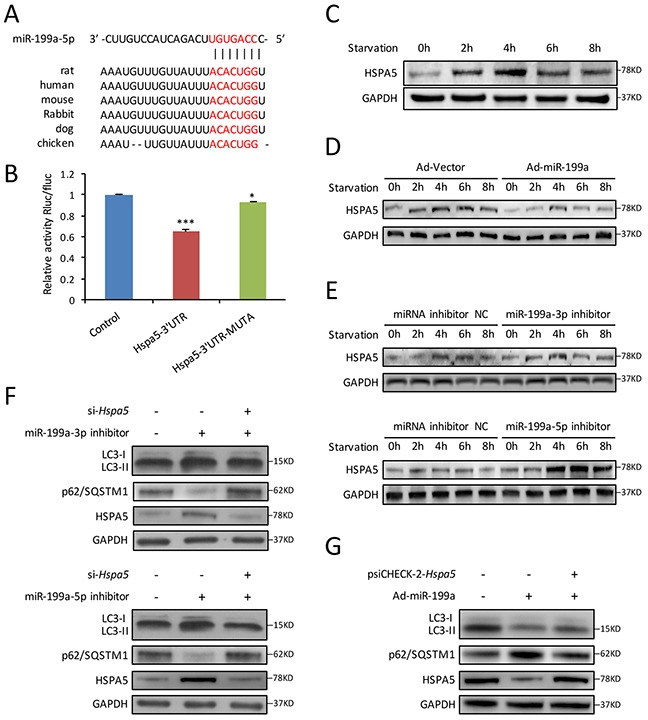
*Hspa5* is identified as the target gene of miR-199a in CMs **(A)** Using Targetscan algorithms, 3′ UTR of *Hspa5* harbored a potential binding site of miR-199a-5p, which was conserved among different species. **(B)** Luciferase assay showed that overexpression of miR-199a-5p in 293T cells could significantly suppress the luciferase activity of a reporter fused with 3′UTR of *Hspa5* mRNA. In cells transfected with psiCHECK-2-*Hspa5* 3′UTR-Mutant, there was less significant difference in the relative luciferase activity between cells treated with miR-199a-5p mimic and cells treated with a negative control. *Dunnett's t* tests were used for comparison between groups. (*P<0.05, ***P<0.001 versus control, n=3). **(C)** Western blot shows HSPA5 protein levels in starved CMs were increased during the first 4 hours, then decreased after 6 h. **(D)** Forced overexpression of miR-199a in CMs significantly decreased the intracellular HSPA5 expression levels. **(E)** Knockdown of endogenous miR-199a-3p or miR-199a-5p markedly increased HSPA5 protein levels as shown by western blot. **(F)** Cardiomyocytes were co-tranfected with miR-199a-3p inhibitor or miR-199a-5p inhibitor and si-*Hspa5*. Knockdown of either miR-199a-3p or miR-199a-5p could promote LC3-II accumulation, p62/SQSTM1 degradation and HSPA5 protein expression after starvation for 4 h. Knockdown of *Hspa5* counteracted the above effects. **(G)** Western blot showed that overexpression of *Hspa5* recovered the inhibitory effect of miR-199a in starvation-induced CMs autophagy. GAPDH was used as endogenous control.

To further explore the mechanism by which miR-199a suppressed starvation-induced autophagy, we searched for potential targets of miR-199a. Computational prediction of targets by TargetScan (www.targetscan.org) identified *Hspa5* as a miR-199a-5p target gene. *Hspa5* is reported to be required for stress-induced autophagy [22]. As shown in Figure 5A, the 3′ UTR of *Hspa5* harbored a potential binding site of miR-199a-5p. Luciferase reporter assay indicated that miR-199a-5p significantly inhibited the luciferase activity in the reporter vector containing wild-type 3′UTR of *Hspa5*. However, the inhibitory effect was weaker in the mutant 3′UTR vector (Figure 5B). The results demonstrated the specificity of miR-199a-5p on *Hspa5* 3′UTR targeting.

For exploring the relationship between miR-199a and *Hspa5* in CMs autophagy, we first detected the variation of HSPA5 protein during CMs autophagy. Western blot showed that HSPA5 expression increased from 2-4 h and then decreased gradually (Figure 5C). Furthermore, we observed that during starvation-induced CMs autophagy, overexpression of miR-199a significantly inhibited *Hspa5* at both protein and mRNA levels (Figure 5D and Supplementary Figure 2A). Conversely, forced inhibition of either miR-199a-3p or miR-199a-5p increased the *Hspa5* protein and mRNA levels in starved CMs (Figure 5E and Supplementary Figure 2B, 2C).

Rescue experiments were performed thereafter. We cotransfected CMs with miR-199a-3p or miR-199a-5p inhibitor together with si-*Hspa5*. The autophagy enhancement induced by miR-199a inhibitor was weakened after *Hspa5* knockdown (Figure 5F). In addition, *Hspa5* was overexpressed from a plasmid lacking of miR-199a response element. Although miR-199a overexpression decreased LC3-II accumulation and p62/SQSTM1 degradation, the miR-199a-mediated suppression of autophagy was reversed upon co-expression of *Hspa5* (Figure 5G). These results demonstrate that *Hspa5* should be a target of miR-199a for autophagy inhibition.

In summary, the present study demonstrated that during starvation-induced CMs autophagy, both miR-199a-3p and miR-199a-5p were down-regulated remarkably. Forced overexpression of miR-199a suppressed CMs autophagy through direct inhibiting *Hspa5*.

## DISCUSSION

In the present study, we introduce miR-199a as an autophagy-related miRNA in CMs. The results reveal that miR-199a serve as a suppressor in starvation-induced CMs autophagy. The expression level of miR-199a under the starvation condition was negatively correlated with activity of autophagy. These results are consistent with the findings of Xu et al [23], who has reported that cisplatin-induced autophagy is associated with decreased levels of the miR-199a-5p in hepatocellular carcinoma cell lines. Yi et al. [24] also found that miR-199a-5p suppressed autophagy in MCF7 cells by inhibiting *DRAM1* and *BECN1*. Most recently, Li et al. [25] reported miR-199a's suppressive role in CMs autophagy during cardiac hypertrophy through down-regulating of *GSK3β* and then activating mTOR.

Here, we identified *Hspa5* as a direct target of miR-199a-5p and subsequently found that overexpression of miR-199a decreased *Hspa5* protein and mRNA levels. Furthermore, *Hspa5* protein and mRNA levels were up-regulated after knockdown of miR-199a-3p or miR-199a-5p. Luciferase reporter assay also confirmed the direct effect of miR-199a-5p on *Hspa5*. More importantly, reintroduction of *Hspa5* in the presence of miR-199a reversed autophagy blockage by this miRNA. Knockdown of *Hspa5* could offset the autophagy activation by miR-199a as well. All these results suggest a direct inhibitory effect of miR-199a on *Hspa5*.

HSPA5 protein, also known as GRP78, is an endoplasmic reticulum (ER) stress associated protein and plays an important role in cellular protection by preventing protein-protein aggregation [22]. Under starvation condition, the environment of protein synthesis in the center of ER changed, following by unfolded or misfolded proteins accumulated and led to ER stress [26, 27]. This phenomenon ultimately induced cell autophagy [28]. HSPA5, which is expressed as an ER stress chaperone synchronously with LC3-II, was associated with autophagy and generally cardiac protection [29, 30]. In this study, we found that expression of HSPA5 was coincidently consistent with LC3-II accumulation and autophagy activation. Moreover, overexpression of *Hspa5* rescued the autophagic inhibitory effect of miR-199a, which suggested the probable role of HSPA5 in CMs autophagy. In Li's research [25], they demonstrated miR-199a impaired CMs autophagy by targeting GSK3β/mTOR complex signaling. It is known that mTOR signaling is a major negative regulatory axis of autophagy [31]. In addition, HSPA5 was reported to activate autophagy through AMPK-mTOR pathway [32, 33]. Specifically, overexpression of *Hspa5* could induce phosphorylation of AMPK and inhibit induction of p-mTOR, which is essential and sufficient to trigger autophagy induction [32, 33]. Combing with our result that miR-199a suppresses CMs autophagy by inhibiting *Hspa5*, AMPK/mTOR pathway could be potentially involved as downstream in regulating CMs autophagy which needs further exploring.

In this study, we demonstrated the relationship between miR-199a and CMs autophagy. In addition, association between miR-199a and cardiac hypertrophy had also been illuminated in our previous study [14]. Actually, miR-199a was shown to be involved in various cardiac events, including myocardial regeneration [34] and hypoxia preconditioning in CMs [35]. In fact, autophagy was described as an important regulator of similar physiological events [36]. Study of the contribution of autophagy-related functions of miR-199a in CMs could provide valuable information about the importance of this miRNA under physiological and pathological conditions.

Moreover, autophagic inhibition by miR-199a may also play important roles *in vivo*, which may provide a basis for the research and development of novel potential therapeutic strategies. However, whether this effect exists in acute ischemic heart *in vivo* remains to be confirmed. In addition, as miRNAs are accepted to regulate multiple target genes, the potential role of other target genes for miR-199a during CMs autophagy could not be excluded. A global analysis of miRNA targets may be needed in the future investigation.

In conclusion, the present study identified miR-199a as a potential regulator of CMs autophagy *in vitro*. Under the starvation condition, down-regulation of miR-199a might be essential for the activation of autophagy through directly promoting *Hspa5*. MiR-199a may be introduced as a potential therapeutic strategy for the protection of CMs in myocardial infarction or ischemic heart disease.

## MATERIALS AND METHODS

### Animals and cell culture

Primary CMs were isolated from neonatal Sprague-Dawley rats (Super-B&K laboratory animal, Shanghai, China) as previously described [14]. The animals were housed in accordance with the National Institutes of Health guide for the care and use of Laboratory animals (NIH Publications No. 8023, revised 1978). The Animal Care and Utilization Committee of the Second Military Medical University (Shanghai, China) approved the study protocols. 293T cells were maintained in the laboratory. Cells were cultured in Dulbecco's modified Eagle medium (DMEM; HyClone, USA) with L-glutamine and 10% fetal bovine serum (FBS; HyClone, USA). Cardiomyocytes were cultured under a starvation condition in EBSS (Gibco, USA) instead of DMEM+10%FBS in order to induce autophagy [15, 16].

### Quantitative real-time polymerase chain reaction (qRT-PCR)

Total RNA was extracted from CMs using Trizol reagent (Invitrogen, USA). Quantitated RNA (500ng) was used for generating cDNA by using M-MLV reverse transcriptase with special stem-loop primer for miRNAs and oligo-dT for mRNAs. Real-time PCR was performed using a standard protocol from the SYBR Green Mix kit (Takara, Dalian, China) on a Rotor-Gene RG-3000A (Corbett Life Science, Sydney, Australia). *U6* small nuclear RNA and *Gapdh* were used as references. Each sample was analyzed in triplicate. The 2^−ΔΔCt^ method was used to determine the relative quantitation of gene expression. Primers used in the amplification reaction were shown in Supplementary Table 1.

### Construction of adenoviruses and cell transfection

Recombinant adenoviruses (Ad) were constructed, amplified and titered, as previously described by Graham and Prevec [37]. All ribosomal DNA was delivered to the CMs via adenovirus vectors using 10 to 20 multiplicity of infection. MiR-199a-3p inhibitor, miR-199a-5p inhibitor, psiCHECK-2-*Hspa5* and si-*Hspa5* were transfected into CMs using Lipofectamine 2000 (Invitrogen, USA) according to the manufacturer's protocol, respectively. The *C.elegans* miRNA inhibitor with similar design and modification method was used as negative control (NC). MicroRNAs and *Hspa5* transfection efficiencies were determined by qRT-PCR.

### Western blot analysis

To assess LC3-I/II, p62/SQSTM1, HSPA5 and GAPDH expression, western blot analysis was carried out on 10% or 15% SDS-PAGE as previously described [38]. The anti-LC3 and anti-GAPDH antibodies were purchased from Medical & Biological Laboratories (MBL, Japan). The anti-HSPA5 antibody was purchased from Abcam (Cambridge, UK). The anti-p62/SQSTM1 antibody was purchased from Abways Technology (Shanghai, China). Anti-rabbit IgG secondary antibody and anti-mouse IgG secondary antibody were purchased from Cell Signaling (Danvers, USA). The dilution ratio of all antibodies was 1:1000. Band intensities were quantified using ImageJ software (NIH, USA).

### Cell immunofluorescence

Cardiomyocytes were fixed with 4% paraformaldehyde for 10 min, permeabilised with 1:1000 Triton/PBS, blocked with 5% goat serum for 30 min at room temperature, and incubated with anti-LC3 antibody (MBL, Japan, dilution 1:200) at room temperature for 1 h. The anti-α-Actinin antibody (dilution 1:300) was purchased from iMedCell Technology (Shanghai, China). After two washes with PBS, the secondary antibody coupled with Alexa Flour 594 (Molecular Probes, Eugene, USA) was added and incubated for 30 min at room temperature. After washing with PBS, the nuclei were stained with 4′,6-diamidino-2-phenylindole (DAPI) and examined under a fluorescence laser scanning confocal TSC-SP5 microscope (Leica, Germany). Intracellular autophagosomes and autolysosomes were probed as LC3 red dots in cytoplasm.

### Transmission electron microscopy

For electron microscopy, CMs were cultured under starvation condition and then treated as previously described [39]. Imaging was done using an H-7650 (HITACHI, Japan) transmission electron microscopy (TEM). Double-membraned autophagic vacuoles or autophagosomes detected under TEM were valid and important for the qualitative analysis of autophagy [15].

### Dual luciferase reporter assay

Luciferase reporter plasmids fused with *Hspa5* 3′UTR/*Hspa5* 3′UTR-Mutant (0.16 μg) and rno-miR-199a-5p mimic (5 pmol) were co-transfected into 293T cells. 48h later, cells were lysed. Luciferase activities were measured using a dual luciferase-reporter assay kit (Promega, USA) on a Lumat LB9507 luminometer (Berthold, Germany). Results were evaluated through normalization of the firefly luciferase activity with renilla luciferase activity as previously described [38].

### Statistical analysis

Data are expressed as mean ± standard deviation (SD) from at least three separate experiments. The differences between groups were analyzed using *t*-tests (for two-group comparison) and analysis of variance (*ANOVA*, for multi-group comparison). *Dunnett's t* tests were used for comparison between groups. Differences were deemed statistically significant at *P*<0.05. Statistical analysis was performed with Statistical Package for Social Sciences (SPSS), version 20.0, for Mac (Chicago, USA).

## SUPPLEMENTARY MATERIALS FIGURES AND TABLE


