# Supplementary material for: MicroRNA-199a acts as a potential suppressor of cardiomyocyte autophagy through targeting Hspa5
Source: Oncotarget. 2017 Jul 10;8(38):63825–34. doi: 10.18632/oncotarget.19133 (PMC5609964; doi:10.18632/oncotarget.19133)
Supplement: Supplementary file 1 [file oncotarget-08-63825-s001.pdf]

# MicroRNA-199a acts as a potential suppressor of cardiomyocyte autophagy through targeting *Hspa5*

## SUPPLEMENTARY MATERIALS

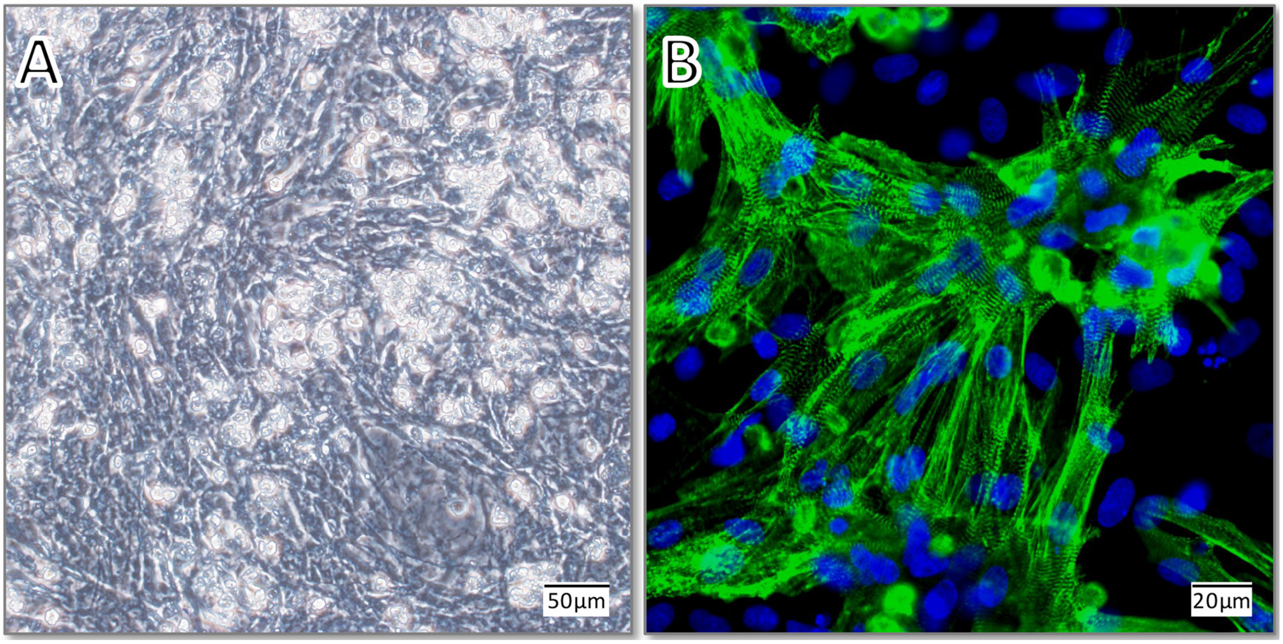


**Supplementary Figure 1: Primary cardiomyocytes isolated from neonatal Sprague-Dawley rats. (A)** Microscopy image of CMs on Day 3. **(B)** Immunofluorescence staining of CMs. α-Actinin (green) indicates CMs. Nuclei were stained with DAPI (blue).

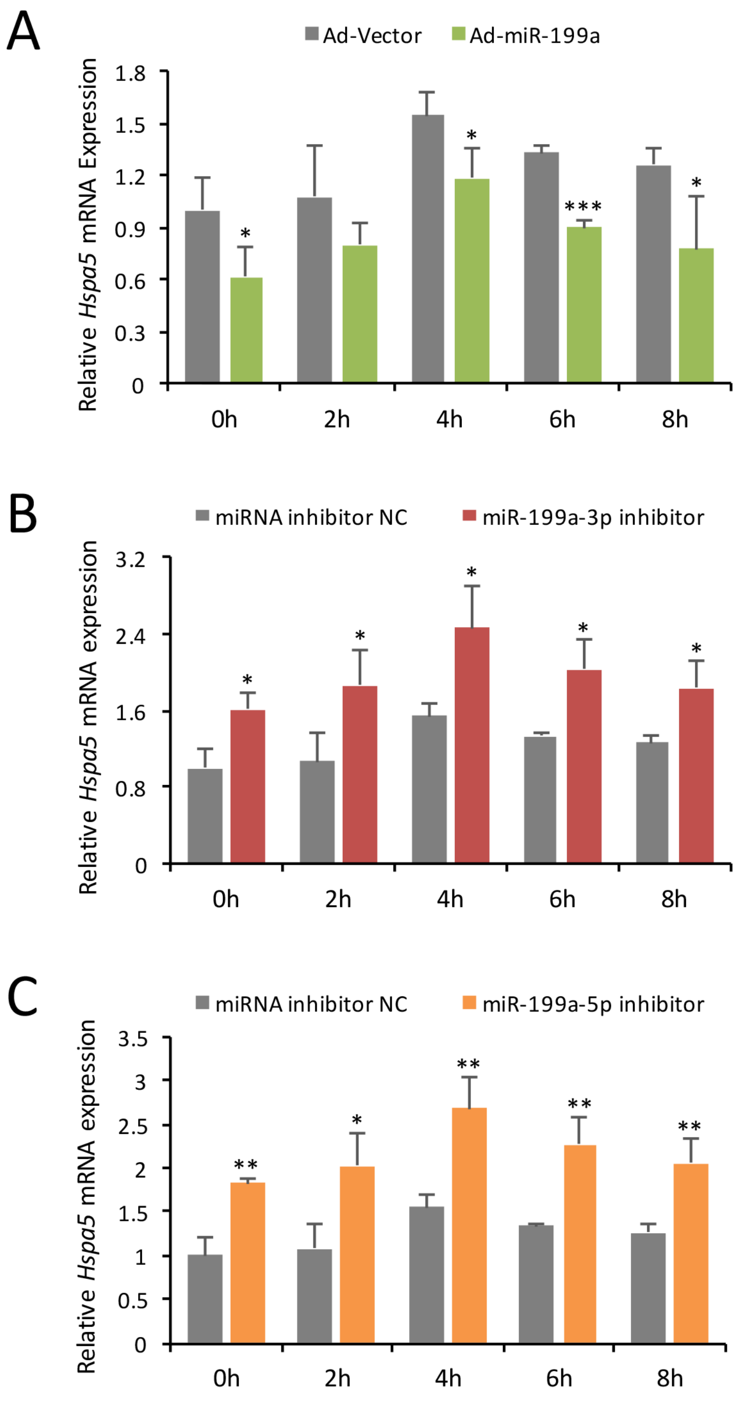


**Supplementary Figure 2: The *Hspa5* mRNA expressions after overexpression or knockdown of miR-199a.** **(A)** Forced overexpression of miR-199a in starved CMs significantly decreased the *Hspa5* expression levels compared with Ad-Vector group at each time point (**P*<0.05, ****P*<0.001, n=3). **(B and C)** Knockdown of either miR-199a-3p or miR-199a-5p in starved CMs markedly increased *Hspa5* expression levels compared with negative control (NC) at each time point (**P*<0.05, ***P*<0.01, n=3). The differences between two groups were analyzed using *t*-tests. *Gapdh* was used as reference.

**Supplementary Table 1: Primers used in the amplification reaction**

| **Primers** | **5’-3’** |
|---|---|
| rno-miR-199a-3p Forward | CTGAGTACAGTAGTCTGCACAT |
| rno-miR-199a-5p Forward | CTGAGTCCCAGTGTTCAGACT |
| miRNAs common Reverse | GTGCAGGGTCCGAGGT |
| rno-*Hspa5* Forward | AACCCAGATGAGGCTGTAGCA |
| rno-*Hspa5* Reverse | ACATCAAGCAGAACCAGGTCAC |
| rno-*Gapdh* Forward | GTCGGTGTCAACGGATTTG |
| rno-*Gapdh* Reverse | ACAAACATGGGGGCATCAG |
| rno-*U6* Forward | CTCGCTTCGGCAGCACA |
| rno-*U6* Reverse | AACGCTTCACGAATTTGCGT |
